# Predicting new cases of hypertension in Swedish primary care with a machine learning tool

**DOI:** 10.1016/j.pmedr.2024.102806

**Published:** 2024-06-30

**Authors:** Anders Norrman, Jan Hasselström, Gunnar Ljunggren, Caroline Wachtler, Julia Eriksson, Thomas Kahan, Per Wändell, Hrafnhildur Gudjonsdottir, Sebastian Lindblom, Toralph Ruge, Andreas Rosenblad, Boel Brynedal, Axel C. Carlsson

**Affiliations:** a Department of Neurobiology, Care Sciences and Society, Division of Family Medicine and Primary Care, Karolinska Institutet, Huddinge, Sweden; b Academic Primary Health Care Centre, Region Stockholm, Stockholm, Sweden; c Division of Biostatistics, Institute of Environmental Medicine, Karolinska Institutet, Stockholm, Sweden; d Division of Cardiovascular Medicine, Department of Clinical Sciences, Danderyd Hospital, Karolinska Institutet, Stockholm, Sweden; e Centre for Epidemiology and Community Medicine, Region Stockholm, Stockholm, Sweden; f Department of Global Public Health, Karolinska Institutet, Stockholm, Sweden; g Womeńs Health and Allied Health Professionals Theme, Karolinska University Hospital, Stockholm, Sweden; h Department of Clinical Sciences Malmö, Lund University & Department of Internal Medicine, Skåne University Hospital, Malmö, Sweden; i Regional Cancer Centre Stockholm-Gotland, Region Stockholm, Stockholm, Sweden; j Department of Medical Sciences, Division of Clinical Diabetology and Metabolism, Uppsala University, Uppsala, Sweden; k Department of Statistics, Uppsala University, Uppsala, Sweden

**Keywords:** Artificial intelligence, Hypertension, Family practice, Gradient boosting, Prediction, Opportunistic screening

## Abstract

**Background:**

Many individuals with hypertension remain undiagnosed. We aimed to develop a predictive model for hypertension using diagnostic codes from prevailing electronic medical records in Swedish primary care.

**Methods:**

This sex- and age-matched case-control (1:5) study included patients aged 30–65 years living in the Stockholm Region, Sweden, with a newly recorded diagnosis of hypertension during 2010–19 (cases) and individuals without a recorded hypertension diagnosis during 2010–19 (controls), in total 507,618 individuals. Patients with diagnoses of cardiovascular diseases or diabetes were excluded. A stochastic gradient boosting machine learning model was constructed using the 1,309 most registered ICD-10 codes from primary care for three years prior the hypertension diagnosis.

**Results:**

The model showed an area under the curve (95 % confidence interval) of 0.748 (0.742–0.753) for females and 0.745 (0.740–0.751) for males for predicting diagnosis of hypertension within three years. The sensitivity was 63 % and 68 %, and the specificity 76 % and 73 %, for females and males, respectively. The 25 diagnoses that contributed the most to the model for females and males all exhibited a normalized relative influence >1 %. The codes contributing most to the model, all with an odds ratio of marginal effects >1 for both sexes, were dyslipidaemia, obesity, and encountering health services in other circumstances.

**Conclusions:**

This machine learning model, using prevailing recorded diagnoses within primary health care, may contribute to the identification of patients at risk of unrecognized hypertension. The added value of this predictive model beyond information of blood pressure warrants further study.

## 1 Background

Hypertension is of major global health concern with a prevalence of 35–50 % in the adult population and a significant contribution to morbidity and mortality (Zhou et al., 2021).

A major issue is that about half of the individuals with hypertension are unrecognized (Zhou et al., 2021). This emphasizes the need for better screening to offer proper management and to reduce the risk of future cardiovascular disease and mortality (Mancia et al., 2023). Since few countries have the resources to offer population based systematic blood pressure screening, opportunistic screening during health care visits is recommended, at least among people with a higher likelihood of elevated blood pressure (Mancia et al., 2023).

Artificial intelligence (AI) holds the potential to improve the detection of hypertension (Chaikijurajai et al., 2020, Tsoi et al., 2021). However, limited evidence exists for its clinical use, and further research is necessary before implementation in health care (Padmanabhan et al., 2021). A recent review aimed to assess the use of machine learning, a type of AI, in predicting hypertension, and highlighted the potential benefits of using AI in predicting hypertension (Silva et al., 2022). Thus, AI-based methods may have the potential to identify additional, previously unknown, factors from the routine data collected by primary care centres, which could help identify individuals at higher risk of hypertension (Silva et al., 2022). The majority of patients with hypertension are identified and monitored through primary care centres; therefore, data from these centres are ideal to study the clinical practice of hypertension (Hasselström et al., 2014).

Our study aimed to utilize machine learning to develop predictive models for diagnosis of hypertension within three years by analysing the diagnostic codes recorded in primary care electronic medical records. The analyses were stratified by sex as there are diverging risk factors and different patterns of diagnosis in males and females (Carlsson et al., 2008, Gerdts et al., 2022).

## 2 Methods

### 2.1 Data sources

The Stockholm Region has a population of approximately 2.4 million. Data for this study was gathered from the Stockholm Region regional health care data warehouse (VAL), which provides information about all health care consultations in primary and secondary care (defined as specialist outpatient care), diagnoses (according to ICD-10), all hospitalisations with diagnoses and procedures, and of sex and age. Cases with a newly recorded diagnosis of hypertension (ICD-10 code I10) were identified across primary and secondary care settings, but the diagnoses used for predicting a new diagnosis of hypertension were exclusively obtained from primary care settings. Up to five controls, individually matched by age and sex to each case were randomly selected from individuals who had not received a diagnosis of hypertension registered in the Stockholm regional health care data warehouse.

The dataset lacked access to recorded systolic or diastolic blood pressure values. Therefore, patient categorization as either cases or controls relied on recorded diagnoses for hypertension (ICD-10 I10). These diagnoses were sourced from primary care records, with no indication provided regarding their accuracy. At the time of the study, the diagnosis and management of hypertension in Swedish health care was recommended to follow the prevailing European Society of Cardiology/European Society of Hypertension guideline (Mancia et al., 2023).

### 2.2 Study design and participants

This study used a case-control design.

The eligible population was individuals aged 30–65 years registered at primary care centres in the Stockholm Region. The prevalence of hypertension, both globally and specifically within a group of countries to which Sweden belongs, classified as “High income western countries,” increases with advancing age and the proportion of individuals with hypertension who remain undiagnosed is lower in the age groups below 65 years (Zhou et al., 2021). Accordingly, the clinical utility the model can provide is expected to be greatest in age groups below 65 years, where the prevalence is not high enough to warrant screening everyone. Thus, we selected 65 years as the upper age limit for inclusion in the study.

Cases and controls were identified from diagnostic codes in medical records during 2014–2019. We included cases and controls who had received at least one other diagnosis at a primary care centre in the Stockholm Region within the three years prior to the diagnosis of hypertension among cases.

For controls, we considered diagnoses registered during the three years prior to the date of the index diagnosis of the case they were matched to. Patients with a diagnosis of hypertension or cardiometabolic disease (i.e., coronary artery disease (I20-I25), atrial fibrillation/flutter (I48), heart failure (I50), stroke (I60-I69, I74, I80), or diabetes mellitus (E10-E14)) recorded 2010–2013 were excluded from the study, as their blood pressure should be part of the regular monitoring required for the underlying condition. Thus, a total of 507,618 individuals were included. These diagnoses (not hypertension), despite being the bases for exclusion of study participants during 2010–2013, may still be registered for both cases and controls during 2014–2019 and subsequently become features utilised in the models.

### 2.3 Feature selection

We went back three years prior to the index date for all participants and listed their diagnoses, based on the top 2000 most common diagnosis codes according to ICD-10 registered in primary care. The diagnoses with at least 50 occurrences were selected, resulting in 1,309 diagnoses as described elsewhere (Wändell et al., 2013).

### 2.4 Data analysis

This study employed the Stochastic Gradient Boosting (SGB) technique for data analysis, a form of machine learning utilized in related research (Friedman, 2001). Tree-based machine learning methods such as SGB have been recommended in a recent *meta*-analysis of machine learning tools for detecting diabetes (Fregoso-Aparicio et al., 2021) and SGB has previously been used to analyse factors influencing lung and colorectal cancer risk (Nemlander et al., 2022).

The SGB models used a Bernoulli loss function fitted to 20,000 trees, each having a maximum depth of five interactions, with a shrinkage (learning rate) of 0.001, a minimum of ten observations in the terminal nodes of the trees, and a subsampling rate (bag fraction) of 0.5. The optimal number of trees to use for prediction was estimated using 10-fold cross-validation, but the model failed to find an optimal number of trees. We were unable to, within a reasonable amount of time, find an optimal number of trees in the model with the most sensitive parameters. Thus, we decided to settle with 20,000 trees, as increasing the number would only marginally improve the model.

We split the data into a 70 % training set and a 30 % test set, ensuring that the proportion of individuals with a recorded diagnosis of hypertension was roughly equal between the training and test data sets.

For each of the two training data sets stratified by sex, we selected diagnoses with at least 100 occurrences, resulting in 346 diagnoses for males and 365 diagnoses for females. The SGB model was then applied to each test dataset to obtain patient-specific probabilities of being diagnosed with hypertension within three. The probabilities that maximized the sum of sensitivity and specificity were used as cut-off values; so that patients with a probability higher than this cut-off were classified as being newly diagnosed with hypertension. The performances of the final models were evaluated using area under the receiver operator characteristics (ROC) curve (AUC), sensitivity, and specificity.

From the SGB model, we obtained a rank of the most important diagnoses related to newly diagnosed hypertension, presented as the normalized relative influence (NRI) score with a corresponding odds ratio of marginal effects (OR_ME_) of being diagnosed with hypertension within three. For each diagnosis, the OR_ME_ was calculated using the probabilities of being newly diagnosed with hypertension obtained using the weighted tree traversal method.

In addition to these SGB models, we tested simplified SGB models to reach the optimal number of trees by splitting the data into a 50 % training set and a 50 % test set. For both genders, the simplified models had a maximum depth of five interactions, a shrinkage (learning rate) of 0.01, a minimum of ten observations in the terminal nodes of the trees, and a subsampling rate (bag fraction) of 0.5.

All analyses were performed in R version 4.2.1 (R Core Team, 2023).

## 3 Results

### 3.1 General

**Fig. 1:**
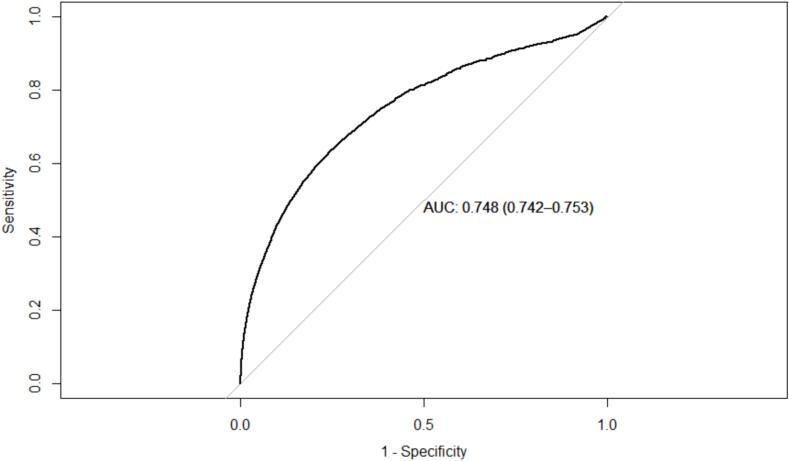
Receiver operator characteristics curve for the optimal stochastic gradient boosting model applied to the females in the test data set.

**Fig. 2:**
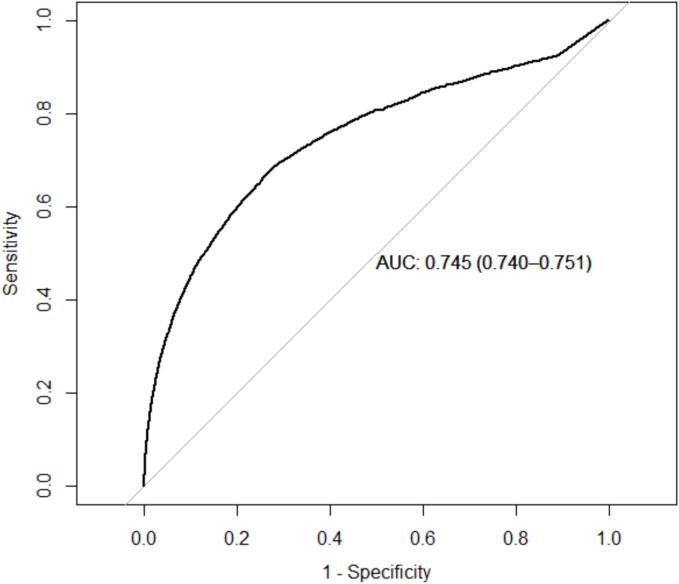
Receiver operator characteristics curve for the optimal stochastic gradient boosting model applied to the males in the test data set.

A total of 84,603 cases and 423,015 controls were included. The training data included 179,714 males and 175,619 females, while the test data included 77,020 males and 75,265 females. The complex SGB model did not reach an optimal number of threes (see method) but showed an AUC of 0.75 (95 % confidence interval (CI) 0.74–0.75) for females and 0.75 (95 % CI 0.74–0.75) for males (Fig. 1, Fig. 2).

The simplified SGB model, where an optimal number of trees was reached, had a poorer performance, showing that our complex model was superior despite not reaching an optimal number of trees (see Supplementary Figs. 1 and 2).

### 3.2 Predictive ability of the SGB model

The predictive ability of the complex SGB model is presented in Table 1, which shows the confusion matrix for males and females based on diagnoses made up to three years prior to the first recorded diagnosis of hypertension in the test dataset. For females, the sensitivity was 63 % and the specificity 76 %, while for males the sensitivity was 68 % and the specificity was 73 %.

**Table 1:** Confusion matrix for predicting presence of registered new hypertension among the 75 265 female and 77 020 male patients in the test dataset using the optimal stochastic gradient.

	Observed		
Predicted	Not Hypertension	Hypertension	Total
Not Hypertension (females)	47 948	4460	52 408
Hypertension (females)	15 251	7606	22 857
Total (females)	63 199	12 066	75 265

Not Hypertension (males)	46 446	4244	50 690
Hypertension (males)	17 351	8979	26 330
Total (males)	63 797	13 223	77 020

Notes: Predictions based on 20,000 trees. Females: sensitivity: 0.630, specificity: 0.759; males: sensitivity: 0.679, specificity: 0.728.

### 3.3 The impact of different ICD-10 codes (in the model)

Among females, 327 diagnoses showed an NRI > 0 %, and 25 of these had an NRI > 1 %. Similarly, among males, 282 diagnoses showed an NRI > 0 %, and 25 of these had an NRI > 1 %. Most of the diagnoses with an NRI > 1 % had an OR_ME_ > 1, See Supplementary Tables 1 and 2. Tables 2a and 2b present the 25 diagnoses with the highest NRI according to sex. 20 out of the 25 most common diagnoses overlapped for females and males. All diagnoses in the machine learning model and their respective NRIs are presented for females and males in Supplemental Table 2. For females, the five diagnostic codes with the highest relative influence (NRI) were “obesity” (9.8 %),” dyslipidaemia” (9.5 %), “persons encountering health services in other circumstance” (5.3 %), “encounter for other special examination without complaint, suspected or reported diagnosis” (5.1 %), and “other and unspecified soft tissue disorders, not elsewhere classified” (4.1 %). Similarly, for males, the top five diagnoses with the highest NRI were “dyslipidaemia” at 18.4 %, “obesity” at 9.8 %, “persons encountering health services in other circumstances” at 7.0 %, “diabetes” at 6.6 %, and “encounter for other special examination without complaint, suspected or reported diagnosis”, at 4.2 %.

**Table 2a:** The 25 variables for females with highest normalized relative influence (NRI) for predicting presence of new hypertension among females using the optimal stochastic gradient boosting (SGB) model with 20,000 trees, together with odds ratios for marginal effects (OR_ME_) of hypertension.

**ICD-10 code**	**Description**	**NRI (%)**	**OR_ME_**
E66	Obesity	9.8	4.1
E78	Dyslipidemia	9.5	4.1
Z76	Persons encountering health services in other circumstances	5.3	1.6
Z01	Encounter for other special examination without complaint, suspected or reported diagnosis	5.1	4.2
M79	Other and unspecified soft tissue disorders, not elsewhere classified	4.1	1.3
E11, E13, E14	Diabetes type 2	4.0	2.5
J06	Acute upper respiratory infections of multiple and unspecified sites	3.3	1.3
R05	Cough	3.0	1.5
R51	Headache	3.0	1.8
F43	Reaction to severe stress, and adjustment disorders	3.0	1.6
Z03	Encounter for medical observation for suspected diseases and conditions ruled out	2.4	1.3
R00	Abnormalities of heart beat	2.4	2
F41	Anxiety disorders	2.4	1.5
R73	Elevated blood glucose level	2.1	4
M54	Dorsalgia	1.6	1.2
J45, J46	Asthma	1.6	1.5
J20	Acute bronchitis	1.5	1.5
R07	Pain in throat and chest	1.4	1.6
M25	Other joint disorder, not elsewhere classified	1.4	1.2
R42	Dizziness and giddiness	1.4	1.5
R53	Malaise and fatigue	1.2	1.2
Z00	Encounter for general examination without complaint, suspected or reported diagnosis	1.1	1.3
I20, I21, I23, I24, I25	Coronary heart disease	1.1	2.8
E03, E05	Thyroid disorders	1.1	1.4
Z72	Problems related to lifestyle	1.0	2.1

**Table 2b:** The 25 variables for males with highest normalized relative influence (NRI) for predicting presence of new hypertension among males using the optimal stochastic gradient boosting (SGB) model with 20,000 trees, together with odds ratios for marginal effects (OR_ME_) of hypertension.

**ICD-10 code**	**Description**	**NRI (%)**	**OR_ME_**
E78	Dyslipidemia	18.4	5.6
E66	Obesity	9.8	6.9
Z76	Persons encountering health services in other circumstances	7.0	1.9
E11, E13, E14	Diabetes type 2	6.6	2.9
Z01	Encounter for other special examination without complaint, suspected or reported diagnosis	4.2	4.2
R73	Elevated blood glucose level	2.6	4.7
Z03	Encounter for medical observation for suspected diseases and conditions ruled out	2.5	1.4
I20, I21, I23, I24, I25	Coronary heart disease	2.5	2.6
M79	Other and unspecified soft tissue disorders, not elsewhere classified	2.4	1.4
R51	Headache	2.2	2.2
G47	Sleep disorders	1.8	1.6
M10	Gout	1.7	2.6
N18, N19, N05	Chronic renal disease	1.6	5.5
J06	Acute upper respiratory infections of multiple and unspecified sites	1.6	1.3
M54	Dorsalgia	1.5	1.2
R07	Pain in throat and chest	1.4	1.7
R06	Abnormalities of breathing	1.3	1.8
R42	Dizziness and giddiness	1.3	1.8
M25	Other joint disorder, not elsewhere classified	1.2	1.3
Z72	Problems related to lifestyle	1.2	2.3
F41	Anxiety disorders	1.1	1.4
R00	Abnormalities of heart beat	1.1	2.1
F90	Attention-deficit hyperactivity disorders	1.1	4.2
R05	Cough	1.1	1.4
F43	Reaction to severe stress, and adjustment disorders	1.0	1.5

### 3.4 Marginal effects

The results for the sex-stratified models showed that the 5 diagnoses with the highest NRI all had an OR_ME_ > 1. For females, these diagnoses were “obesity” (OR_ME_ = 4.1), “dyslipidaemia” (OR_ME_ = 4.1), “encounter for other special examination without complaint, suspected or reported diagnosis” (OR_ME_ = 4.2), “encounters with health services in other circumstances” (OR_ME_ = 1.6), “and other and unspecified soft tissue disorders, not elsewhere classified” (OR_ME_ = 1.3). Similarly, for males, the top 5 diagnoses were “obesity” (OR_ME_ = 6.9), “dyslipidaemia” (OR_ME_ = 5.6), “encounter for other special examination without complaint, suspected or reported diagnosis” (OR_ME_ = 4.2), “diabetes type 2” (OR_ME_ = 2.9), and “encounters with health services in other circumstances” (OR_ME_ = 1.9).

## 4 Discussion

This study applied an SGB model to predict whether an individual had hypertension or not, based on data on previous recorded diagnostic codes in primary care electronic medical records from the three years preceding a registered diagnosis of hypertension. In individuals 30–65 years old with no known major cardiovascular disease or diabetes, the model showed a modest sensitivity and specificity of 63 % and 76 % in females, and 68 % and 73 % in males, respectively. The diagnostic codes contributing the most to the model irrespective of sex, with the highest NRI were “dyslipidaemia”, “obesity”, and “encounters with health services in other circumstances”, where “encounter for issue of repeat prescription” is the most common subcode.

### 4.1 Results in perspective

The results of this study demonstrate an association between several diagnostic codes and recorded diagnosis of hypertension within three years, aligned with previous research and AI models. Specifically, overweight or obesity (Kanegae et al., 2020, Liao et al., 2022), high fasting glucose (Kanegae et al., 2020, Liao et al., 2022), dyslipidaemia (Sakr et al., 2018, Ye et al., 2018), previously higher blood pressures (Kanegae et al., 2020, Sakr et al., 2018), coronary heart disease (Ye et al., 2018), multiple chronic diseases (Ye et al., 2018) and psychiatric diseases (Ye et al., 2018) all exhibit a strong association in this model. The results in this study are in accordance with the well-established association with risk factors within the metabolic syndrome, where high blood pressure occurs alongside with glucose intolerance and diabetes mellitus type 2, dyslipidaemia, abdominal obesity, and microalbuminuria (Carlsson et al., 2009). Previous studies developed AI models to detect high blood pressure in undiagnosed patients, but only a few were conducted specifically within primary care settings (Silva et al., 2022).

Other diagnoses that predict hypertension are those indicating that the patient has visited health care for any reason, for example renewal of prescription. Common diagnoses in primary care of various types, such as “Other and unspecified soft tissue disorders” (including myalgia (Wändell et al., 2013) and dorsalgia (Wändell et al., 2013), and acute respiratory tract infections (Wändell et al., 2013), also had OR_ME_:s >1 for both females and males. The most likely explanation for these results is that the likelihood of having hypertension detected increases in individuals with many visits to primary care centres and that individuals with these diagnoses visit primary care centres more.

Values for sensitivity, specificity, and AUC ROC in the prediction model used in this study show modest results. Previous prediction models have relied on blood pressure measurements (Sheppard et al., 2016). Previous studies with access to blood pressure measurements have AUC ROC between 0.766 and 1.00 (Ford et al., 2013, Silva et al., 2022). This study, however, is based on receiving a diagnosis of hypertension in the electronic medical records and adds new complementary information. In this study, we demonstrate that prediction of diagnosis of hypertension within three years in possible based solely on previously recorded diagnoses without the need for collecting other clinical information.

### 4.2 Strengths and limitations

One strength of this study was the inclusion of all new cases of a recorded diagnosis of hypertension in Region Stockholm during the study period, providing a detailed dataset of previously registered diagnoses for machine learning analysis. Conducting the study in a real-world primary care setting, where more than 50 % of all healthcare encounters take place (Wändell et al., 2013) enhances the external validity of the findings and their applicability to clinical practice. Another strength is that we excluded patients with hypertension, cardiovascular disease, and diabetes for three years (2010–2013) prior to finding diagnosis of hypertension within three years (2014–2019). Yet, these diagnoses occurred as risk factors if they presented before the hypertension diagnosis during the study period.

A weakness of the study design is that the model depends only on previous diagnoses registered by the general practitioner. Previous studies have highlighted that general practitioners may not document all symptom diagnoses presented during consultations (Ford et al., 2013), and the registration of diagnoses may vary over time. In addition, registration of related diseases like obesity, dyslipidaemia, chronic kidney disease, and peripheral arterial disease are known to be limited in primary care in Sweden (Wändell et al., 2013). Given the known low detection rate of hypertension it is likely that several controls have undetected hypertension. Further, ethnicity, which plays a significant role in the prevalence of hypertension, could not be included, as data on ethnicity are not registered in the clinical setting (Carlsson et al., 2008).

Relying solely on diagnosed hypertension rather than actual blood pressure values introduces uncertainty regarding the results, as patients with elevated blood pressure readings where diagnoses have not been made are not classified as cases. A sensitivity analysis for the number of visits was not performed and we cannot determine whether the NRI for certain diagnoses is linked to the diagnosis itself or to the fact that the patient has visited the primary care and received a diagnosis. Additionally, the model has a weakness in its clinical utility in that patients without previous visits have not been included in the model.

Machine learning techniques such as SGB do not make any distributional assumptions about the underlying data (Chen and Guestrin, 2016). However, risks can arise from the environmental constraints set during the design of the study, such as the age limit of 65 years, which may exert an influence and limit the generalizability of the results. One strength lies in the algorithm's consistent interpretation of diagnoses once they have been established. However, there is a risk that bias in the initial assignment of diagnoses may influence the reliability of the model.

### 4.3 Clinical implications

International guidelines recommend that people at high risk of having elevated blood pressure should be offered opportunistic screening for hypertension in relation to health care visits, and patients with established hypertension should have their blood pressure controlled at least annually (Mancia et al., 2023). Swedish recommendations align with these guidelines. In Sweden, most adults visit primary care, for various reasons, at least once every year. Although this would offer an excellent chance for opportunistic screening, blood pressure is not routinely measured at primary care visits in Swedish primary care. Further, a recent Swedish study on the time required to follow guidelines for the management of hypertension suggests that there is room for improvement (Johansson et al., 2023). We postulate that the modelling approach used in our machine learning model could assist clinicians in flagging patients at-risk for hypertension and ensure that a blood pressure check is offered by the care giver. This may help optimize resource and time utilization, and eventually increase the number of patients with previously undetected hypertension being offered appropriate treatment.

Further studies could include additional variables or qualitatively assessing how stakeholders in primary care perceive the model (Terry et al., 2022). Additional studies targeting older individuals are also warranted.

## 5 Conclusion

The study demonstrated that a machine learning model using diagnostic codes from electronic medical records could predict a diagnosis of hypertension within three years better than chance. The top contributing diagnoses were consistent with known risk factors for hypertension.

## Declaration of Generative AI and AI-assisted technologies in the writing process

During the preparation of this work the author(s) used ChatGPT 3.5 for linguistic accuracy. After using this tool/service, the author(s) reviewed and edited the content as needed and take(s) full responsibility for the content of the publication.

## Funding

Karolinska Institutet Research Foundations (Stockholm, Sweden; 2022–02092), and by grants from the Swedish state under the agreement between the Swedish government and the county council in Stockholm, SLL-NSV (990472).

## Ethical approval

The study was approved by the Swedish Ethical Review Authority and need for individual informed consent was waived. All data were pseudonymized to protect patient privacy. The data in the present study are available for research purposes, after ethical approval, at halsodata.rst@regionstockholm.se.

## CRediT authorship contribution statement

**Anders Norrman:** Writing – original draft. **Jan Hasselström:** Writing – review & editing. **Gunnar Ljunggren:** Writing – review & editing. **Caroline Wachtler:** Writing – review & editing, Conceptualization. **Julia Eriksson:** Writing – review & editing, Formal analysis, Data curation, Conceptualization. **Thomas Kahan:** Writing – review & editing. **Per Wändell:** Writing – review & editing, Conceptualization. **Hrafnhildur Gudjonsdottir:** Writing – review & editing. **Sebastian Lindblom:** Writing – review & editing. **Toralph Ruge:** Writing – review & editing. **Andreas Rosenblad:** Writing – review & editing, Visualization, Methodology, Formal analysis, Conceptualization. **Boel Brynedal:** Writing – review & editing. **Axel C. Carlsson:** Writing – review & editing, Project administration, Methodology, Conceptualization.

## Declaration of competing interest

The authors declare that they have no known competing financial interests or personal relationships that could have appeared to influence the work reported in this paper.

## Data Availability

Data will be made available on request.
