# Supplementary material for: Predicting new cases of hypertension in Swedish primary care with a machine learning tool
Source: Prev Med Rep. 2024 Jun 30;44:102806. doi: 10.1016/j.pmedr.2024.102806 (PMC11292513; doi:10.1016/j.pmedr.2024.102806)
Supplement: Supplementary Data 1 [file mmc1.docx]

Supplementary Figures. Predicting New Cases of Hypertension in Primary Care: A Machine Learning Tool

**
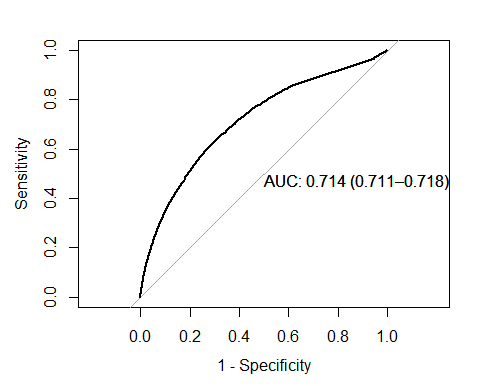
**

Supplementary figure 1. Receiver operator characteristics curve for the optimal stochastic gradient boosting model applied to the men in the test data set.


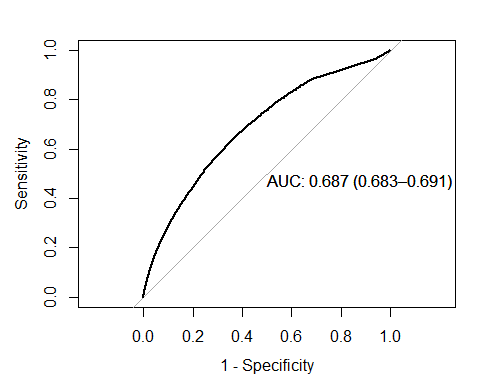


Supplementary figure 2. Receiver operator characteristics curve for the optimal stochastic gradient boosting model applied to the women in the test data set.
